# Pre-Transplant Expression of CCR-2 in Kidney Transplant Recipients Is Associated With the Development of Delayed Graft Function

**DOI:** 10.3389/fimmu.2022.804762

**Published:** 2022-03-16

**Authors:** Paola Pontrelli, Simona Simone, Federica Rascio, Francesco Pesce, Francesca Conserva, Barbara Infante, Giuseppe Castellano, Fabio Sallustio, Marco Fiorentino, Gianluigi Zaza, Anna Gallone, Michele Battaglia, Pasquale Ditonno, Giovanni Stallone, Loreto Gesualdo, Giuseppe Grandaliano

**Affiliations:** ^1^ Department of Emergency and Organ Transplantation, Nephrology and Urology Units, University of Bari Aldo Moro, Bari, Italy; ^2^ Department of Medical and Surgical Sciences, Renal Unit, University of Foggia, Foggia, Italy; ^3^ Department of Clinical Sciences and Community Health, Nephrology Unit, University of Milano and Fondazione Cà Grande Ospedale Maggiore Policlinico, Milano, Italy; ^4^ Department of Internal Medicine, University of Bari Aldo Moro, Bari, Italy; ^5^ Department of Basic Medical Sciences, Neurosciences and Sense Organs, University of Bari Aldo Moro, Bari, Italy; ^6^ Department of Translational Medicine and Surgery, Nephrology Unit, Università Cattolica del Sacro Cuore and Fondazione Policlinico Universitario “A. Gemelli”, Rome, Italy

**Keywords:** kidney transplantation, delayed graft function, gene expression, peripheral blood mononuclear cells, CCR-2

## Abstract

**Background:**

Delayed graft function (DGF) leads to a reduced graft survival. Donors’ features have been always considered as key pathogenic factors in this setting. The aim of our study was to evaluate the recipients’ characteristics in the development of DGF.

**Methods:**

We enrolled 932 kidney graft recipients from 466 donors; 226 recipients experienced DGF. In 290 donors, both recipients presented with early graft function (EGF, group A), in 50 both recipients experienced DGF (group B), and in 126 one recipient presented with DGF and the other with EGF (group C). In group C, we selected 7 couples of DGF/EGF recipients and we evaluated the transcriptomic profile by microarray on circulating mononuclear cells harvested before transplantation. Results were validated by qPCR in an independent group of 25 EGF/DGF couples.

**Findings:**

In the whole study group, DGF was associated with clinical characteristics related to both donors and recipient. In group C, DGF was significantly associated with body mass index, hemodialysis, and number of mismatches. In the same group, we identified 411 genes differently expressed before transplantation between recipients discordant for the transplant outcome. Those genes were involved in immune dysfunction and inflammation. In particular, we observed a significant increase in DGF patients in the expression of C–C chemokine receptor type 2 (CCR2), the monocyte chemoattractant protein-1 (MCP-1) receptor. CCR-2 upregulation was confirmed in an independent cohort of patients.

**Conclusions:**

Our results suggest that recipients’ clinical/immunological features, potentially modulated by dialysis, are associated with the development of DGF independently of donors’ features.

## Introduction

Delayed graft function (DGF) is a form of post-transplant acute kidney injury, commonly defined as the requirement for dialysis in the recipient within 7 days after renal transplantation (1). Although the incidence depends on its definition and by donor type, DGF is reported in up to 50% of renal allografts (2, 3).

An increasing body of evidence suggests that DGF might adversely affect short- and long-term transplant outcomes, increasing the risk of acute rejection and reducing both graft and patient survival with a consequent increase in healthcare costs (4–6).

Several immunological and non-immunological factors can influence DGF onset and graft loss, and those factors can be referred to donors and recipients features or, in alternative, to the transplant procedure itself (7). The use of expanded criterion donors and prolonged warm or cold ischemia time are significantly associated with the development of DGF (8). Indeed, ischemia-reperfusion damage, with the subsequent activation of pro-inflammatory and pro-fibrotic pathways, and donors’ features have been always considered as the pivotal pathogenic factors in this setting (9, 10).

Several predictive biomarkers (11, 12) and predictive models have been proposed to quantify the risk of DGF using different methods such as logistic regression or machine learning (13). These methods comprise a combination of donor risk factors, including age, body weight, and kidney function, variables related to the surgical procedure (for example, cold ischemia time), and recipient risk factors, such as obesity, diabetes, and dialysis-related variables (2, 14). However, the importance of the recipients’ characteristics, in particular their immune-phenotype, in the development of DGF is not yet clearly defined, since there is a lack of knowledge of the recipient characteristics that could be mechanistically involved in the development of DGF.

Thus, the aim of our study was to evaluate the role of recipients’ characteristics, especially the immune phenotype by the analysis of gene expression profiling in peripheral blood mononuclear cells (PBMC), that could be mechanistically involved in the pathogenesis and development of DGF.

## Materials and Methods

### Patients and Ethics

This is a single-center, observational, prospective, cohort study performed in a University Hospital. We enrolled 932 uremic patients receiving a kidney transplant from 466 deceased donors with brain death in the University of Bari Kidney Transplant Center from January 1, 1999, to December 31, 2011. The clinical and research activities being reported are consistent with the Principles of the Declaration of Istanbul. The study was approved by the Local Ethical Committee (Prot. N. 670/CE-2017) and was in accordance with the Declaration of Helsinki. We excluded from the study patients receiving a double kidney transplant, graft recipients with primary non-function, and patients who received a single kidney from donors whose other kidney was transplanted in a different center. The main demographic and clinical data of the patients included in the study are summarized in **Table 1**.

**Table 1:** The main demographic and clinical data of the patients included in the study.

	All (%)	EGF (%)	DGF (%)	p
n.	932	706 (75.7)	226 (24.3)	
Age (years)	45.8 ± 11.2	44.9 ± 11.3	48.5 ± 10.7	<0.0001
Gender (M/F)	595 (63.8)/337 (36.2)	444 (62.9)/262 (37.1)	150 (66.4)/76 (33.6)	0.4
Dialysis (HD/PD)	851 (91.3)/81 (8.7)	631 (89.4)/75 (10.6)	218 (96.5)/8 (3.5)	0.005
Dialysis vintage (years)	6.2 ± 4.4	5.8 ± 4.3	7.4 ± 4.7	<0.0001
HCV+ (n,%)	156, 16.7	98, 13.9	58, 25.4	<0.0001
Body mass index (BMI) (kg/m^2^)	24.0 ± 3.8	23.7 ± 3.8	24.9 ± 3.8	0.0001
Panel reactive antibodies (%)	7.5 ± 5.2	7.4 ± 4.9	7.6 ± 5.7	0.8
Cold ischemia time (hours)	12.7 ± 5.0	13.0 ± 5.2	12.2 ± 6.5	0.4
Mismatches (n)	3.2 ± 0.9	3.1 ± 0.9	3.3 ± 0.9	0.1
Donor age (years)	45.1 ± 17.5	43.3 ± 17.3	50.5 ± 16.7	<0.0001
Donor serum creatinine (mg/dl)	1.28 ± 0.75	1.23 ± 0.6	1.44 ± 1.0	0.04
Cause of death (trauma/vascular)	304 (32.6)/628 (67.4)	289 (40.9)/417 (59.1)	75 (33.2)/151 (66.8)	0.02
Cyclosporine (n)	408 (43.7)	307 (43.5)	101 (44.7)	0.6
Tacrolimus (n)	479 (51.4)	368 (52.1)	111 (49.1)	0.8
mTOR inhibitors (n)	80 (4.9)	76 (4.4)	4 (6.2)	<0.0001

None of the grafts underwent machine perfusion. All patients included in the study received anti-CD25 monoclonal antibodies at day 0 and day 4 after transplantation as induction therapy. Maintenance immunosuppression was represented by calcineurin inhibitors, either cyclosporine or tacrolimus, mycophenolic acid, and corticosteroids. A small group of patients received mTOR inhibitors.

To exclude patients who were dialyzed for reasons other than impaired graft function (post-transplant hyperkalemia, fluid retention, etc.), DGF was defined as the need of more than one session of dialysis in the first week after transplantation (15). The patients who did not present these features were included in the EGF groups.

Among the 466 donors, in 290 couples of recipients both patients presented with early graft function (EGF, group A), in 50 couples both recipients experienced DGF (group B), and in 126 one recipient presented DGF and the other promptly recovered graft function (group C).

### PBMC Isolation, RNA Extraction, and Microarray

Twenty ml of whole blood was harvested from patients included in the study at the time of transplantation, before the administration of induction therapy. PBMCs were isolated by density separation over a Ficoll-Paque™ (GE Healthcare, Uppsala, Sweden). In group C, we randomly selected seven EGF/DGF couples for microarray analysis (training group). To validate the microarray results, we randomly chose in the same group C further 25 kidney graft recipients with EGF and 25 with DGF (testing group). The main demographic characteristics of patients included in the microarray cohort and in the testing group are reported in **Table 2**.

**Table 2:** The main demographic and clinical characteristics of patients included in the microarray group and in the testing group.

	Training (microarray) group		Testing group	
	EGF (%)	DGF (%)	EGF (%)	DGF (%)
n.	7	7	25	25
Age (years)	46.8 ± 11.0	47.8 ± 9.3	46.8 ± 11.0	47.8 ± 9.3
Gender (M/F)	4 (57.1)/3 (42.9)	5 (71.4)/2 (28.6)	16 (64)/9 (36)	17 (68)/8 (32)
Dialysis (HD/PD)	5 (71.4)/2 (28.6)	6 (85.7)/1 (14.3)	21 (84)/4 (16)	22 (88)/3 (12)
Dialysis vintage (years)	5.8 ± 5.0	7.3 ± 4.9	6.0 ± 4.9	7.5 ± 5.3
HCV+ (n)	1 (14.3)	1 (14.3)	2 (8)	3 (12)
Body mass index (BMI) (kg/m^2^)	22.8 ± 4.3	24.9 ± 4.8	23.7 ± 5.3	25.0 ± 5.9
Panel reactive antibodies (%)	6.5 ± 6.9	7.0 ± 8.7	7.7 ± 6.2	7.5 ± 6.9
Cold ischemia time (hours)	12.9 ± 6.4	13.1 ± 6.5	13.0 ± 6.3	13.2 ± 6.6
Mismatches (n)	3.4 ± 1.1	3.5 ± 1.2	3.3 ± 1.3	3.5 ± 1.0
Cyclosporine (n)	2 (28.6)	2 (28.6)	4 (16)	4 (16)
Tacrolimus (n)	4 (57.1)	5 (71.4)	21 (84)	21 (84)
mTOR inhibitors (n)	1 (14.3)	0	0	1

Total RNA was extracted in the selected patients, using the RNeasy Mini Kit (Qiagen AG, Basel, Switzerland), and qualitatively and quantitatively analyzed through Agilent Bioanalyzer (Agilent Technologies, Santa Clara, CA). Only samples with good quality, as indicated by a RIN > 8, were used in the microarray experiment.

For the microarray experiments, we used the GeneChip^®^ Human Genome U133A oligonucleotide microarray (Affymetrix, Santa Clara, CA) which contains 22,283 gene probe sets, representing 12,357 human genes, plus approximately 3,800 expressed sequence tag clones (ESTs), according to the manufacturer’s instructions. We used the default settings of Affymetrix Microarray Suite software version 5 (MAS 5.0; Affymetrix) to calculate scaled gene expression values.

Results of the microarray experiments are available in the database of the European Bioinformatics Institute (EMBL-EBI) and are accessible through Experiment ArrayExpress accession E-MTAB-10747.

### Real-Time PCR

We validated the results gathered by microarray in the testing group by quantitative real time-PCR. Reverse transcription of total RNA (500 ng) was performed using the High-Capacity cDNA Reverse Transcription Kit (Applied Biosystems, Foster City, CA), following the manufacturer’s instructions. Relative quantification was obtained as previously described (16) by a comparative Ct method using 18s rRNA as a stably expressed endogenous reference gene. The following TaqMan Gene Expression Assays (Applied Biosystems) were employed: Hs00704702_s1 (CCR2), Hs00234140_m1 (MCP1), and Hs01060665_g1 (ACTB). The qPCR was carried out with the Roche Light-Cycler Real-Time PCR system with 5 µl TaqMan Universal PCR Master Mix in a 10-µl-reaction volume.

### Statistical Analysis

Data are presented as mean ± standard deviation (SD) or median and interquartile range and compared by ANOVA or Wilcoxon test, as appropriate. The multivariate logistic regression model was used to identify the variables independently associated with DGF. The risk is expressed as odds ratio (OR) - 95% confidence interval (CI). In the multivariate analysis, all the variables that at the univariate analysis presented p ≤ 0.1 were included. p < 0.05 was considered statistically significant. Statistical analysis was performed with the Statistical Package for Social Sciences version 23.0 (SPSS, Inc., Chicago, IL).

The differentially expressed genes obtained by microarray experiments were identified by applying a fold change ≥1.5 and p value <0.05 after comparison of the two groups by t-test (moderate t-test). Permutation analysis was applied to reduce the false discovery rate. Results were statistically analyzed using the software GeneSpring GX 12.5 in order to identify genes differentially expressed, and functionally analyzed using the Ingenuity Pathway Analysis (IPA) software (www.ingenuity.com) as previously reported (16, 17).

## Results

### Donors’ and Recipients’ Characteristics Predict DGF in the Whole Cohort

We prospectively enrolled 932 kidney graft recipients from 466 donors for both kidneys, 226 of whom experienced DGF. Patients with DGF were significantly older, more frequently on renal replacement therapy with hemodialysis, with significantly longer dialysis vintage, higher prevalence of HCV infection, and higher BMI than patients with EGF. Donors of patients with DGF were older, with a worse renal function, and more frequently with a cerebrovascular cause of death (**Table 1**). The multivariate analysis revealed that donors’ age and renal function as well as recipients’ features, including dialysis vintage, BMI, and HCV infection, were independently associated with the development of DGF (**Table 3**).

**Table 3:** The univariate and multivariate analyses of donors’ and recipients’ features associated with DGF.

	Univariate			Multivariate		
	OR	95% CI	p	OR	95% CI	p
Recipient age (years)	1.030	1.016–1.044	<0.0001	0.978	0.942–1.016	0.2
Dialysis vintage (years)	1.075	1.035–1.117	0.0002	1.096	1.013–1.185	0.02
Body mass index (BMI) (kg/m^2^)	1.080	1.038–1.125	0.0002	1.136	1.035–1.248	0.007
Recipient’s HCV (positive vs. negative)	2.110	1.462–3.044	<0.0001	2.829	1.242–6.446	0.01
HD (vs. PD)	3.249	1.537–6.869	0.002	1.317	0.396–4.385	0.6
Donor’s age (years)	1.025	1.015–1.034	<0.0001	1.032	1.006–1.058	0.01
Donor’ serum creatinine (mg/dl)	1.384	1.007–1.932	0.04	1.645	1.098–2.464	0.01
Cause of death (cerebrovascular vs. trauma)	1.479	1.059–2.065	0.02	1.013	0.455–2.258	0.9

Interestingly, among the 466 donors included in the present study, in the 62% of the cases (290) both recipients experienced a prompt recovery of graft function (group A). Only in 10.7% (50 cases) did both recipients develop DGF (group B), whereas in 27% (126 cases) the two recipients presented a discordant recovery of graft function (group C). The three groups significantly differ for donors’ features. In particular, donors of group A presented the most favorable characteristics in terms of age, cause of death, and renal function, whereas donors of group C presented with a worse clinical profile (**Table 4**). This observation underlines the relevance of recipients’ risk factors in group C in addition to the ones related to donors.

**Table 4:** The main clinical and demographic characteristics of Groups A, B, and C.

	Group A (%)	Group B (%)	Group C (%)	p
n.	580	100	252	
Age (years)	44.6 ± 11.4	49.0 ± 12.0	47.3 ± 10.1	<0.0001
Gender (M/F)	365 (62.9)/215 (37.1)	69 (69)/31 (31)	161 (63.9)/91 (36.1)	0.5
Dialysis (HD/PD)	523 (90.1)/57 (9.9)	95 (95)/5 (5)	232 (92.1)/20 (7.9)	0.2
Dialysis vintage (years)	5.8 ± 4.3	7.0 ± 4.5	6.8 ± 4.5	0.01
HCV+ (n,%)	76, 13.1	24, 24.0	56, 22.2	0.0006
Body mass index (BMI) (kg/m2)	23.8 ± 3.8	24.8 ± 3.8	24.2 ± 3.6	0.03
Panel reactive antibodies (%)	7.1 ± 4.3	7.3 ± 4.4	7.0 ± 5.1	0.9
Cold ischemia time (hours)	13.2 ± 5.0	10.6 ± 3.2	13.1 ± 5.4	<0.0001
Mismatches (n)	3.1 ± 0.9	3.0 ± 0.8	3.4 ± 0.9	<0.0001
Donor age (years)	42.3 ± 17.3	53.0 ± 15.9	48.5 ± 16.9	<0.0001
Donor serum creatinine (mg/dl)	1.18 ± 0.61	1.69 ± 1.16	1.37 ± 0.84	0.002
Cause of death (trauma/vascular)	211 (36.4)/304 (63.6)	24 (24)/66 (66)	81 (32.1)/150 (67.9)	0.02
Cyclosporine (n)	300 (51.7)	93 (93)	15 (5.9)	<0.0001
Tacrolimus (n)	262 (45.2)	3 (3)	214 (84.9)	<0.0001
mTOR inhibitors (n)	74 (3.1)	4 (4)	2 (0.8)	<0.0001

### The Relevance of Recipients’ Clinical Features in the Incidence of DGF

We, then, analyzed the clinical data of the 252 recipients of groups C, where from each donor we have one recipient who developed DGF and one who did not (**Table 5**). The univariate analysis showed that patients with DGF presented a significantly longer time on dialysis and higher BMI and were more frequently on hemodialysis than on peritoneal dialysis compared with patients with EGF (**Table 6**). In addition, HCV prevalence and a higher number of mismatches were associated with DGF, although this association did not reach statistical significance. The frequency of right versus left kidney was equally distributed between the two groups (66 left and 60 right kidneys in the EGF group and 60 left and 66 right kidneys in the DGF group). At the multivariate logistic regression, the variables that remain significantly and independently associated with DGF were BMI, number of mismatches, and renal replacement therapy with hemodialysis (**Table 6**).

**Table 5:** The main clinical and demographic features of Group C: EGF and DGF patients.

	Group C (%)	EGF (%)	DGF (%)	P
n.	252	126	126	
Age (years)	47.3 ± 10.1	46.8 ± 11.0	47.8 ± 9.3	0.4
Gender (M/F)	161 (63.9)/91 (36.1)	80 (63.5)/46 (36.5)	81 (64.3)/45 (35.7)	0.9
Dialysis (HD/PD)	232 (92.1)/20 (7.9)	109 (86.5)/17 (13.5)	123 (97.6)/3 (2.4)	0.0006
Dialysis vintage (years)	6.8 ± 4.5	5.9 ± 4.1	7.7 ± 4.8	0.008
HCV+ (n,%)	56, 22.2	22, 17.4	34, 26.9	0.07
Body mass index (BMI) (kg/m2)	24.2 ± 3.6	23.5 ± 3.3	24.8 ± 3.9	0.007
Panel reactive antibodies (%)	7.0 ± 5.1	7.5 ± 5.9	7.3 ± 6.7	0.9
Cold ischemia time (hours)	13.1 ± 5.4	13.0 ± 5.3	13.2 ± 5.5	0.8
Mismatches (n)	3.4 ± 0.9	3.3 ± 0.9	3.5 ± 0.9	0.1
Cyclosporine (n)	15 (5.9)	19 (15.1)	19 (15.1)	1
Tacrolimus (n)	214 (84.9)	107 (84.9)	107 (84.9)	1
mTOR inhibitors (n)	2 (0.8)	2 (1.5)	2 (1.5)	1

**Table 6:** Univariate and multivariate analyses of the features of Group C associated with DGF.

	Univariate			Multivariate		
	OR	95% CI	p	OR	95% CI	p
Dialysis vintage (years)	1.094	1.021–1.172	0.01	1.047	0.967–1.132	0.2
Body mass index (BMI) (kg/m^2^)	1.102	1.025–1.184	0.008	1.154	1.051–1.268	0.002
Recipient’s HCV (positive vs. negative)	1.747	0.954–3.200	0.07	1.530	0.697–3.356	0.3
HD (vs. PD)	6.394	1.824–22.415	0.003	4.699	1.169–18.886	0.03
Mismatches (n)	1.206	0.917–1.588	0.1	1.661	1.153–2.392	0.006

### Recipients Who Developed DGF Showed Transcriptomic Profiles Featuring a Specific Immune Signature in Peripheral Blood Mononuclear Cells

**Figure 1:**
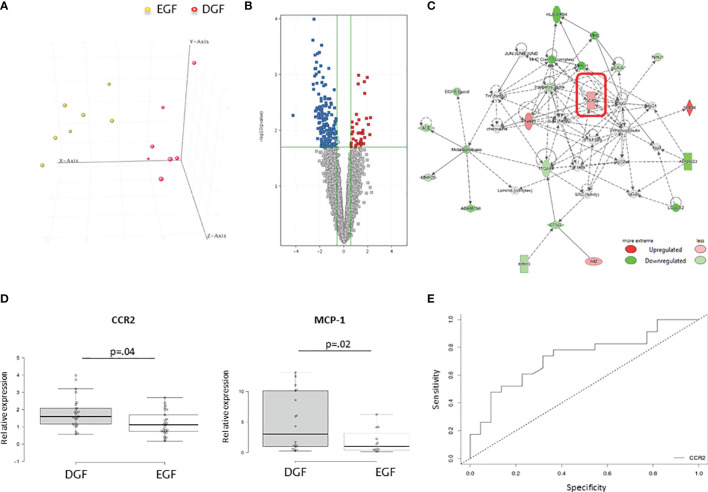
**(A)** Principal component analysis (PCA) of mRNAs discriminating DGF and EGF subjects. DGF (n = 7) and EGF (n = 7) patients are indicated in red and yellow, respectively. **(B)** Volcano plot of the 411 differentially expressed genes among DGF patients and EGF patients with a FDR <0.05 and a FC > 1.5. **(C)** Functional network of the differentially expressed genes among DGF patients and EGF controls. The most significant network is displayed graphically as nodes (genes) and edges (biological relationship between nodes). The node color intensity indicates the fold change of that gene’s expression. Shaded nodes are those genes identified by our microarray analysis, and empty nodes represent genes automatically included by IPA. The shapes of nodes indicate the functional class of the gene product, and the lines indicate the type of interaction. **(D)** Validation by quantitative real-time PCR of CCR2 and MCP-1 in PBMCs isolated from an independent group of 25 DGF and 25 EGF patients for CCR-2 gene expression and 11 DGF and 11 EGF for MCP-1 gene expression. Data are expressed as median and 25th and 75th percentiles in boxes and 5th and 95th percentiles as whiskers. **(E)** ROC curve analysis to evaluate the sensitivity and specificity of CCR2 as a predictive marker of DGF.

In order to identify recipients’ transcriptomic patterns associated with DGF, we analyzed gene expression profiling of PBMCs at the time of transplantation in 7 recipients who developed DGF and 7 who had EGF after transplantation paired from the same donor, randomly selected from group C, since patients belonging to this group exclude all the bias linked to donor. The analysis of the transcriptomic profiles, applying a FC ≥1.5, showed that 411 genes were differentially expressed in PBMC before transplantation in graft recipients who experienced a DGF compared to those who had a normal functional recovery of the graft. Principal component analysis (PCA) revealed a complete separation of the two patient groups based on the expression profiles of the 411 differentially regulated genes (**Figure 1A**).

The analysis by IPA of the main biological and pathological functions in which the differentially expressed genes (**Figure 1B**) were included indicated inflammatory disease (p range = 1.3E-03-1.5E-2, 33 genes) and inflammatory response (p range = 2.65E-03-1.07E-02, 49 genes), two functional patterns featuring the immunological profile of hemodialysis patients.

IPA analysis also revealed the presence of 5 upstream regulators (liraglutide, EOGT, PKA, CCL2, NPS), which are molecules that can affect the expression, transcription, or phosphorylation of other molecules included in the dataset according to interactions described in literature. Interestingly, among this there was CCL-2 with an activation z-score of 1.206 (the z-score algorithm is used to make predictions and is designed to reduce the possibility that random data will generate significant predictions) (18) and a p-value of 8.06E-04. Among the 9 molecules interacting with CCL-2 and included in the dataset (IFNAR1, CCR2, FOS, FOLR2, CD40, NCF1, IL23A, SCARB1, BIRC5) identified by IPA software, there was CCR2, with an expression fold change in DGF vs. EGF of 1.869. The main functional networks in which the differentially expressed genes were included demonstrated the existence of several networks potentially modulated in patients who developed DGF. CCR-2 was included among the highest-ranked network (IPA score = 20) associated with inflammatory response and cell-to-cell signaling and interaction (**Figure 1C**). We decided to focus on this molecule, since our group already demonstrated that an increased CCR2 gene and protein expression on uremic peripheral blood mononuclear cells may contribute to chronic micro-inflammation related to dialysis (19). Thus, we aimed to investigate whether CCR-2-increased expression might also influence DGF occurrence. CCR2 or CD192 is a seven-transmembrane G-protein-coupled receptor that interacts with several ligands, in particular monocyte chemoattractant protein 1 (MCP-1), a chemokine which specifically mediates the chemotaxis of monocytes/macrophages.

### CCR2 Might Represent a Predictive Marker of DGF

To confirm the microarray data and to evaluate whether the identified genes were specific for recipients who developed DGF after kidney transplantation, quantitative real-time PCR was used to compare the expression levels of both CCR-2 and MCP-1 in an independent group of PBMC from recipients who developed DGF (*n* = 25) and those who presented EGF after transplantation (*n* = 25). We confirmed that before transplantation, recipients who developed DGF had higher levels of both CCR2 and MCP-1 (**Figure 1D**). In addition, we observed a significantly higher CCR2 expression in HD compared with PD patients (HD 1.66 ± 0.83 vs. PD 0.71 ± 0.32; p = 0.003) and a significant and direct correlation of CCR2 gene expression with dialysis vintage (r2 = 0.117, p = 0.2).

Finally, we applied a ROC curve analysis to evaluate the sensitivity and specificity of CCR2 as a predictive marker of DGF. The AUC was 0.732, and the value of relative expression of CCR2 of 1.38 allowed discriminating patients who developed DGF with a specificity of 68% and a sensitivity of 74% (**Figure 1E**).

## Discussion

The present study provides for the first time an integrated overview of the recipients’ characteristics that may influence DGF occurrence after kidney transplantation and describe a molecular signature based on whole-genome PBMC gene expression profiles of those recipients who will develop DGF compared to those who will have a normal graft function recovery after kidney transplantation.

Doshi et al., examining recipients’ pairs from the UNOS database, who shared a common deceased donor and were discordant in DGF occurrence, identified recipients’ factors including gender, race, diabetes, and obesity that were strongly associated with DGF risk (20). In line with these results, we observed that BMI, number of mismatches, and hemodialysis were independent predictors of DGF in pairs of recipients from the same donor. It is well known that ischemia reperfusion injury may induce a strong recipient immune response (21, 22) and can represent an important factor for long-term allograft failure. However, compared to Doshi’s dataset (20), our patients’ population presented a limited cold ischemia time, due to the regional allocation policy currently in use in Italy. Thus, it is conceivable that the short cold ischemia time featuring our study reduced the role of this variable on DGF incidence rate. In addition, Doshi et al. (20) performed their analysis on a population of patients identified based on the definition of DGF as the need of dialysis during the first week after transplantation and on a smaller group where DGF was identified using the definition that we adopted in the present study. The definition of DGF as the need of dialysis during the first week after transplantation might result in the inclusion of patients with a normal recovery of graft function that may undergo a dialysis session for a transient volume overload immediately after transplantation or for the appearance of a significant hyperkalemia.

The recipients’ immunological features, possibly modulated by dialysis modality (hemodialysis vs. peritoneal dialysis), are significantly associated with the development of DGF. A lower number of recipients’ pre-transplant circulating regulatory T cells were predictive of delayed or slow graft function in kidney graft recipients from deceased donors (23). In order to understand the role of the recipients’ immune system in the pathogenesis and development of DGF, we analyzed specific gene expression profiles of circulating immune cells. Early transcriptomic biomarkers identified with this approach might represent valuable mechanistic information, suggesting a possible molecular mechanism explaining the association of DGF with recipients’ clinical features and might help clinicians to identify patients with higher risk and suggest potential novel therapeutic approaches.

In the present study, we report that DGF occurrence was associated with specific recipient gene expression profiles of PBMC at the time of transplantation, mainly involving the inflammatory pathway. This molecular observation might fit with the clinical data suggesting hemodialysis as an independent risk factor for DGF when compared with peritoneal dialysis. Indeed, we have demonstrated by a similar microarray-based approach that the expression profiles of circulating lymphomonocytes of hemodialysis patients were characterized by the upregulation of the inflammatory pathway when compared with peritoneal dialysis patients that resembles the one observed in graft recipient with DGF (24). Peritoneal dialysis has been often recognized as a protective factor for DGF, although the cause of the potential beneficial effect of this dialysis modality was identified in the frequent overhydration of peritoneal dialysis patients facilitating the recovery of diuresis after transplantation.

Among the genes characterizing the inflammatory signature predicting DGF, CCR2 expression might represent an interesting molecular candidate. Chemokine production is a characteristic feature occurring after ischemia-reperfusion injury and is associated with an increased risk of acute and chronic allograft rejection (25). Increased MCP-1 expression at the tissue and urinary level is a predictor of acute kidney injury and is associated with an adverse outcome after graft rejection (26, 27). Moreover, several inflammatory cytokines such as G-CSF, IL-6, IL-9, IL-16, and MCP-1 are released by the kidney from brain dead donors, thus initiating an inflammatory state of the graft and massive inflammatory cytokine release upon reperfusion (28). High levels of these cytokines could therefore promote the recall of monocyte/macrophages in the graft, thus representing a promoting factor for the development of DGF. This evidence strongly supports our results of an increased expression of CCR2 before transplantation in the PBMCs of DGF patients compared to graft recipients presenting with an early graft function.

Patients with chronic renal failure undergoing hemodialysis treatment have elevated serum levels of MCP-1 and an increased expression of its CCR2 receptor on circulating monocytes (29). This process is secondary to both uremia (30) and the activation of the coagulation cascade induced by the contact between the blood and the dialysis membranes (19). Indeed, we have previously demonstrated that the activation of the coagulation cascade during hemodialysis may induce CCR2 gene and protein expression in circulating lymphomonocytes. Interestingly, this increased expression was significantly reduced by an approach that limited coagulation cascade priming on the dialyzer (19). Moreover, Liu et al. (31) have already demonstrated that ischemia reperfusion injury promotes production of IL-18 from endothelial cells and expands a T-cell population (CD4+CD45RO+PD-1hiICOS+CCR2+CXCR5-) displaying features of recently described T peripheral helper cells with increased CCR2 expression.

In accordance with this evidence, in our predictive model DGF occurrence was strictly associated with the number of mismatches and hemodialysis and could depend on the specific expression of the MCP-1/CCL2 receptor CCR2 on PBMC, whose increased levels could promote the recruitment of recipient inflammatory cells in the graft, thus influencing the onset of DGF.

Our data would suggest that the measurement of CCR2 expression before transplantation on the recipients alongside the evaluation of the clinical characteristics of the donor and recipient (32) could be used to develop novel predictive models of DGF, although this hypothesis should be formally confirmed in larger prospective studies. The association between an increased CCR2 gene expression and DGF might represent valuable mechanistic information, suggesting a possible molecular mechanism explaining the association of DGF with recipients’ clinical features and, in particular, with previous hemodialysis treatment. This approach would allow implementing new therapeutic strategies in patients at higher risk of DGF such as the allocation of organs with a reduced ischemia time or from optimal donors to patients at risk of DGF; the use of reperfusion machines after organ harvesting; and the inhibition of inflammation both in the donor and in the recipient. In addition to these therapeutic approaches, our data would suggest that also MCP-1 antagonists might represent an interesting alternative in the attempt to prevent DGF and improve long-term graft outcomes.

The main limitation of our investigation is its being a single-center study, being well known that single-center studies result in a selection bias. However, we tried to overcome this limit in the transcriptomic study by randomly selecting the patients included in the testing and validation groups. In addition, a strength of our study is represented by the prospective analysis.

In conclusion, our data suggest that recipients’ clinical and immunological features, possibly modulated by dialysis, independently of donors’ characteristics, are significantly associated with the development of DGF. In addition, we identified potential transcriptomic biomarkers for DGF that might be introduced in clinical practice to define the risk for DGF before kidney transplantation.

## Data Availability Statement

The datasets presented in this study can be found in online repositories. The names of the repository/repositories and accession number(s) can be found in the following: https://www.ebi.ac.uk/arrayexpress/, E-MTAB-10747.

## Ethics Statement

The studies involving human participants were reviewed and approved by the Policlinico di Bari Ethical Committee (Prot. N. 670/CE-2017) and were in accordance with the Declaration of Helsinki. The patients/participants provided their written informed consent to participate in this study.

## Author Contributions

PP and SS conceived and performed the experiments and clinical analysis, designed and coordinated the study, analyzed the data, and drafted the manuscript. FR and FC carried out the experiments, analyzed the data, and revised the manuscript. FP collected the clinical data, helped to interpret the results, and revised the manuscript. FS supported the analysis of microarray data. BI, GC, MF, GZ, and GS enrolled the patients, participated in the performance of the research, and revised the manuscript. AG, MB, and PD participated in the research design and revised the manuscript. LG and GG designed, coordinated, and supervised the study and critically revised the manuscript. All authors contributed to the article and approved the submitted version.

## Conflict of Interest

The authors declare that the research was conducted in the absence of any commercial or financial relationships that could be construed as a potential conflict of interest.

## Publisher’s Note

All claims expressed in this article are solely those of the authors and do not necessarily represent those of their affiliated organizations, or those of the publisher, the editors and the reviewers. Any product that may be evaluated in this article, or claim that may be made by its manufacturer, is not guaranteed or endorsed by the publisher.

## Abbreviations

CCR2, C–C chemokine receptor type 2; DGF, delayed graft function; EGF, early graft function; MCP-1, monocyte chemoattractant protein-1; PBMC, peripheral blood mononuclear cells.
